# Electrical field stimulation-induced contractions on *Pantherophis guttatus* corpora cavernosa and aortae

**DOI:** 10.1371/journal.pone.0196123

**Published:** 2018-04-19

**Authors:** Rafael Campos, Fabíola Z. Mónica, Alberto Fernando Oliveira Justo, José Carlos Cogo, Edvana de Toledo Oliveira, Ronilson Agnaldo Moreno, Edson Antunes, Gilberto De Nucci

**Affiliations:** 1 Faculty of Medical Sciences, Department of Pharmacology, University of Campinas, (UNICAMP), Campinas, Brazil; 2 Faculty of Biomedical Engineering, Brazil University, Itaquera—São Paulo, Brazil; 3 University of Vale do Paraiba (UNIVAP), São José dos Campos, Brazil; 4 Institute of Biomedical Sciences, University of Sao Paulo (USP), Sao Paulo, Brazil; University of Calgary, CANADA

## Abstract

A tetrodotoxin (TTX)-resistant mechanism is responsible for the electrical field stimulation (EFS)-induced contractions and relaxations of *Crotalus durissus terrificus* corpora cavernosa. Here it was investigated whether this mechanism also occurs in corpora cavernosa and aortae of the non-venomous snake *Pantherophis guttatus* corpora cavernosa and aortae. Corpora cavernosa and aortic rings isolated from *Pantherophis guttatus* snake were mounted in organ bath system for isometric tension recording. EFS-induced contractions in both tissues were performed in the presence and absence of guanethidine (30 μM), phentolamine (10 μM) and tetrodotoxin (1 μM). In another set of experiments, the endothelium was removed from aortic rings and EFS-induced contractions were performed in the denuded rings. Electrical field stimulation-induced contractions were frequency-dependent in *Pantherophis guttatus* corpora cavernosa and aortic rings. The contractions were significantly reduced in the presence of guanethidine (30 μM) or phentolamine (10 μM). Pre-treatment with tetrodotoxin had no effect on the EFS-induced contractions of either corpora cavernosa or aortic rings. Surprisingly, the EFS-induced contractions of aortic rings denuded of endothelium were almost abolished. These results indicate that the TTX-resistant mechanism is present in EFS-induced contractions of *Pantherophis guttatus* corpora cavernosa and aortae. The experiments performed in the aorta indicate that the endothelium is the main source for the release of catecholamines induced by EFS.

## Introduction

A tetrodotoxin-insensitive electrical field stimulation (EFS) is responsible for both relaxations [1] and contractions [2] on *Crotalus durissus terrificus* corpora cavernosa. These results are in contrast with mammalian tissues, since EFS-induced contractions of rabbit corpora cavernosa [3] or relaxations in human corpora cavernosa [4] are abolished by TTX pre-treatment. Use of A-803467, an inhibitor of TTX-resistant sodium channel had no effect in the EFS-induced contractions of *Crotalus durissus terrificus* corpora cavernosa, suggesting a possible non-adrenergic terminal source for the catecholamine release in this tissue. Although tyrosine hydroxylase enzyme was identified in the corpus cavernosum nerve terminals of rabbits [5], monkeys [2] and humans [6], it was only observed by immunohistochemisty in the endothelium of *Crotalus durissus terrificus* corpora cavernosa, indicating the endothelium as a possible source for the catecholamine release. Here it is demonstrated that the same TTX-resistant mechanism is present in both corpora cavernosa and aortic rings of the non-venomous snake *Pantherophis guttatus*. Furthemore, the finding that the endothelium removal of the aortic rings almost abolished EFS-induced contractions further corroborates the concept that the endothelium is the main source of catecholamine release in these tissues.

## Material and methods

### Animals

All experimental procedures using *Pantherophis guttatus* were approved by the Institutional Animal Care and Use Committee (Committee for Ethics in the Use of Animal—CEUA/UNICAMP: protocol number 3949–1), and were performed in accordance with the Ethical Principles for Animal Research adopted by the Brazilian College for Animal Experimentation.

*Pantherophis guttatus* (body weight: 400–750g; male and female) were provided by the Serpentarium of the Center for the Study of Nature at the University of Vale do Paraiba (UNIVAP, São José dos Campos, SP, Brazil).

### Chemical and reagents

Acetylcholine, phenylephrine, phentolamine, prazosin, guanethidine, sodium nitroprusside, N(G)-Nitro-L-arginine methyl ester and tetrodotoxin were purchased from Sigma-Aldrich Chemicals Co. (Missouri, USA).

### Tissue preparation

The snakes were killed with isoflurane inhalation followed by ketamine (70 mg/kg) administration (intracelomatic route) and their corpora cavernosa and aortae were removed and immediately placed in Krebs- Henseleit solution at 27°C. Subsequently, four strips of corpora cavernosa (10 mm) and aortic rings (3 mm) were obtained and suspended vertically between two metal hooks in 10 mL organ baths containing Krebs-Henseleit solution: (mM) NaCl (118), KCl (4.7), CaCl_2_ (2.5), MgSO_4_ (1.2), NaHCO_3_ (25), KH_2_PO_4_ (1.2) glucose (5.6) gassed with a mixture of 95%O_2_: 5% CO_2_ (pH 7.4) at 27°C [7].

### Functional protocols in *Pantherophis* corpora cavernosa and aortic rings

In aortic rings, following the 45 minute stabilization period, endothelium integrity was evaluated by acetylcholine (1 μM)-induced relaxation. A relaxation exceeding 80% in a ring pre-contracted with phenylephrine (1 μM) was considered as a signal of endothelial functional integrity. In another set of experiments, the endothelium was removed with the aid of a thin stick. The muscular integrity was assessed by a relaxation induced by sodium nitroprusside (SNP; 1 μM).

*Pantherophis guttatus* corpora cavernosa and aortic rings were submitted to electrical field stimulation (EFS)-induced contraction (30 V for 10 seconds and 60 V for 30 seconds, subsequently, at 4–16 Hz in square-wave pulses; 0.5 ms pulse width; 0.2 ms delay) using a Grass S88 stimulator (Astro-Medical, Industrial Park, RI, USA). EFS-induced contractions were performed in the presence and absence of the non-selective alpha-adrenoceptor blocker, phentolamine (10 μM), guanethidine (30 μM), a substance that depletes noradrenaline stores, tetrodotoxin (TTX; 1 μM), a selective sodium channel blocker and in aortic rings with endothelium denuded.

In another set of experiments, *Pantherophis guttatus* aortic rings were pre-contracted with phenylephrine (1 μM) and when a sustained contraction was obtained, concentration-response curves to ACh (100 ρM- 100 μM) were constructed in the presence and absence of the nitric oxide synthase inhibitor N(G)-Nitro-L-arginine methyl ester (L-NAME;100 μM).

### Data analysis

Data are expressed as mean ± standard error of mean (S.E.M) of the number of experiments. To analyze the pharmacological characterization of EFS-induced contractions, two paired contractions in the presence and absence of antagonists were performed, with the first stimulus being the “control” response (mili-Newtons). Concentration-response curves to acetylcholine were expressed as perceptual phenylephrine induced contraction. Student’s t-test (paired or unpaired depending on the protocol) and one–way analysis of variance (ANOVA) were used. A p value < 0.05 was considered significant.

## Results

### Evaluation of adrenergic and sodium-channel involvement in EFS-induced contractions of *Pantherophis guttatus* corpora cavernosa and aortic rings

**Fig 1:**
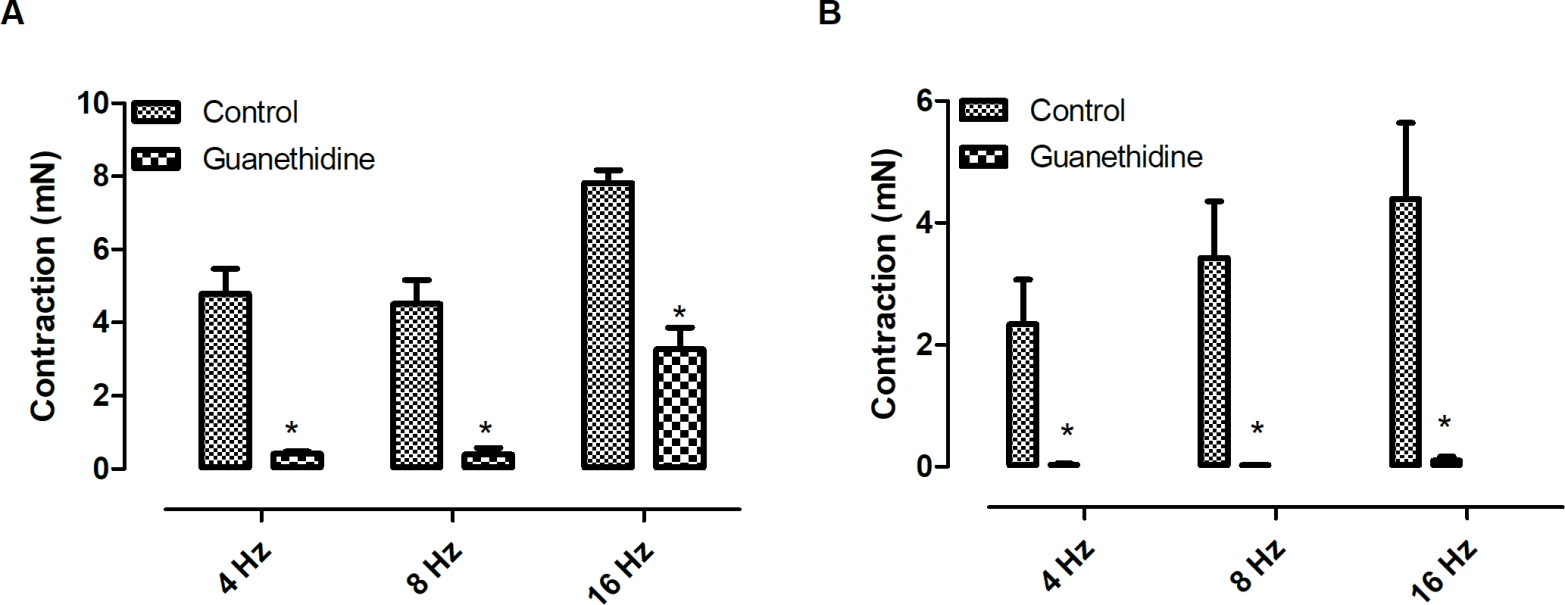
**Effect of guanethidine (30 μM) on EFS-induced contractions on *Pantherophis* corpora cavernosa (A) and aortic rings (B)**. Data are expressed as mean ± S.E.M. *P<0.05 Vs control (paired Student’s t test) (n = 3, for each group).

**Fig 2:**
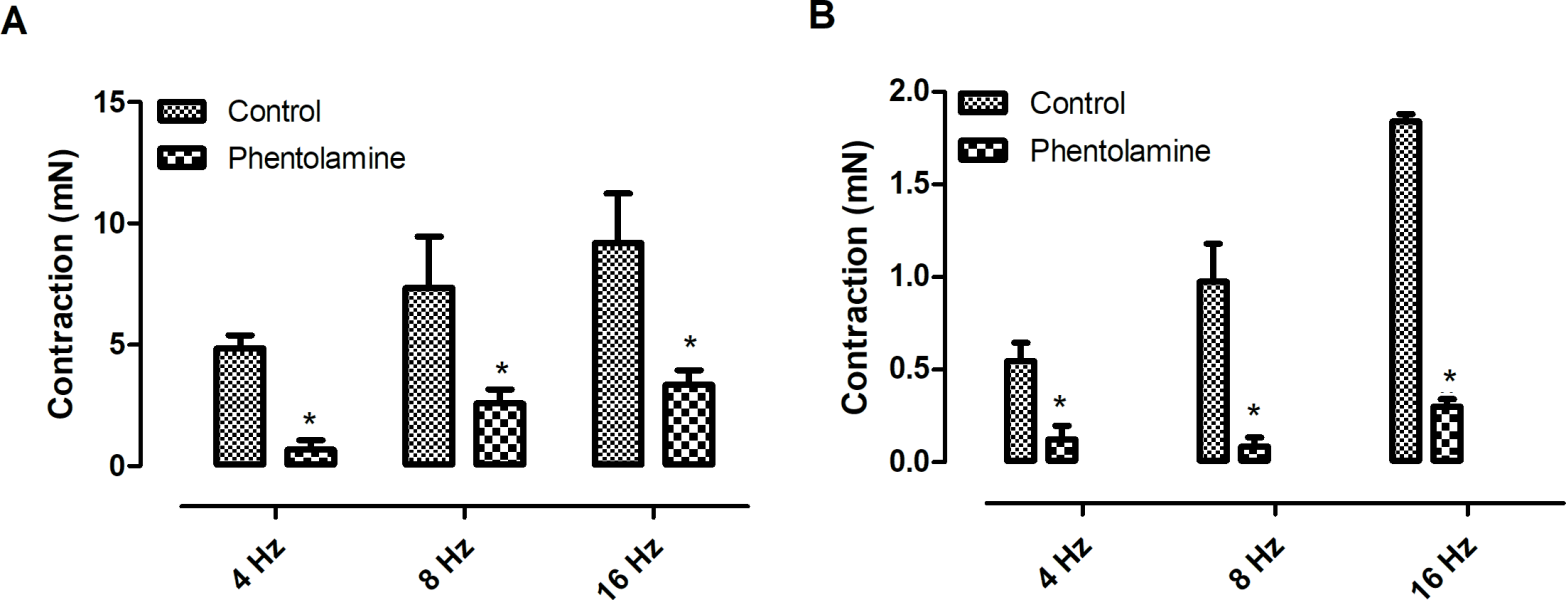
**Effect of phentolamine (10 μM) on EFS-induced contractions of *Pantherophis* corpora cavernosa (A) and aortic rings (B)**. Data are expressed as mean ± S.E.M. (n = 3, for each group). *P<0.05 Vs control (paired Student’s t test).

**Fig 3:**
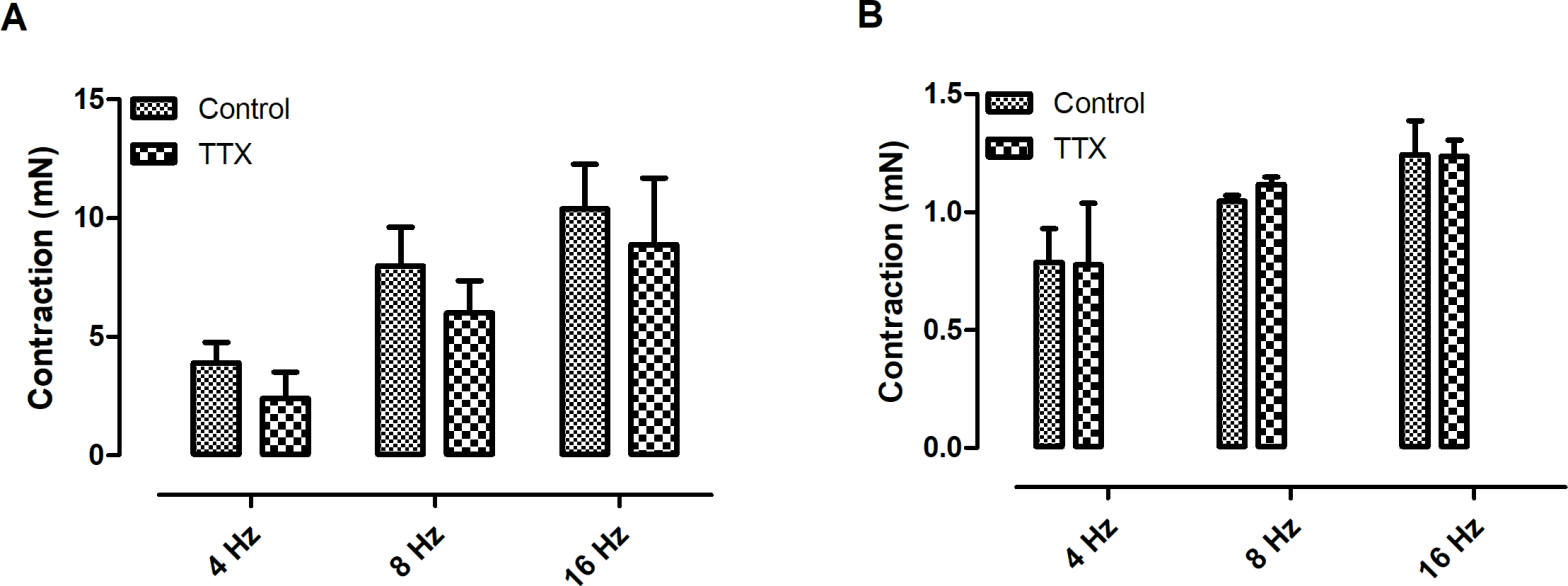
**Effect of tetrodotoxin (TTX; 1 μM) on EFS-induced contractions of *Pantherophis* corpora cavernosa (A) and aortic rings (B)**. Data are expressed as mean ± S.E.M. (n = 3, for each group).

Electrical field stimulation induced frequency-dependent contractions of both *Pantherophis guttatus* corpora cavernosa and aortic rings. In order to investigate the catecholamine participation in EFS–induced contraction, guanethidine (30 μM), an inhibitor of catecholamine release, was added to the bath [2]. Guanethidine reduced the EFS-induced contractions in both tissues (Fig 1A and 1B) (n = 3, for each group) (p < 0.05; paired t-test) suggesting the involvement of catecholamine in this event. The participation of α-adrenoreceptors in the EFS-response, was evaluated incubating the tissue with the α-blocker phentolamine (10 μM) [8]. Similarly to guanethidine, phentolamine also reduced the EFS-induced contractions in both tissues indicating the participation of α-receptors in the EFS-induced contraction. (n = 3, for each group) (p < 0.05; paired t-test) (Fig 2A and 2B). The participation of sodium channel was investigated using the sodium channel blocker tetrodotoxin (1 μM). At this concentration, tetrodotoxin is known to fully block neurogenic response [4,8]. Tetrodotoxin did not alter the response in both tissues (Fig 3A and 3B).

### Role of vascular endothelium in acetylcholine-induced relaxation in *Panterophis* aortic rings

**Fig 4:**
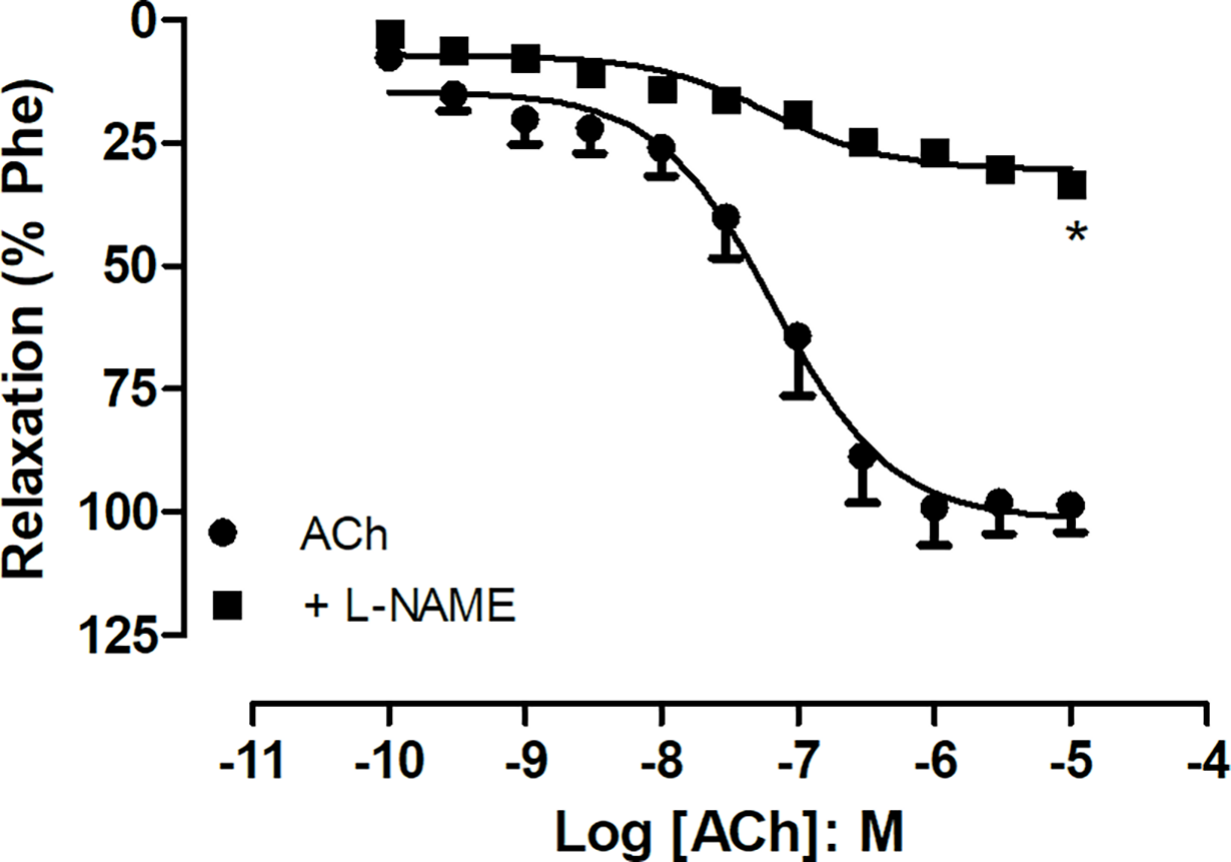
Concentration-response curve to acetylcholine (100 ρM- 10 μM) in the presence and absence of N(G)-Nitro-L-arginine methyl ester (L-NAME; 100 μM). Data are expressed as mean ± S.E.M. *P<0.05 Vs ACh (One-Way ANOVA).

Acetylcholine induced concentration-dependent relaxation in *Pantherophis guttatus* aortic rings (maximal response 101 ± 4.1%) (n = 6). The addition of the nitric oxide synthesis inhibitor L-NAME (100 μM) reduced the ACh response (maximal response 30.4 ± 1.1%) (n = 3) (p< 0.05; ANOVA) (Fig 4) [9].

### Role of vascular endothelium on EFS-induced contractions in *Panterophis* aortic rings

**Fig 5:**
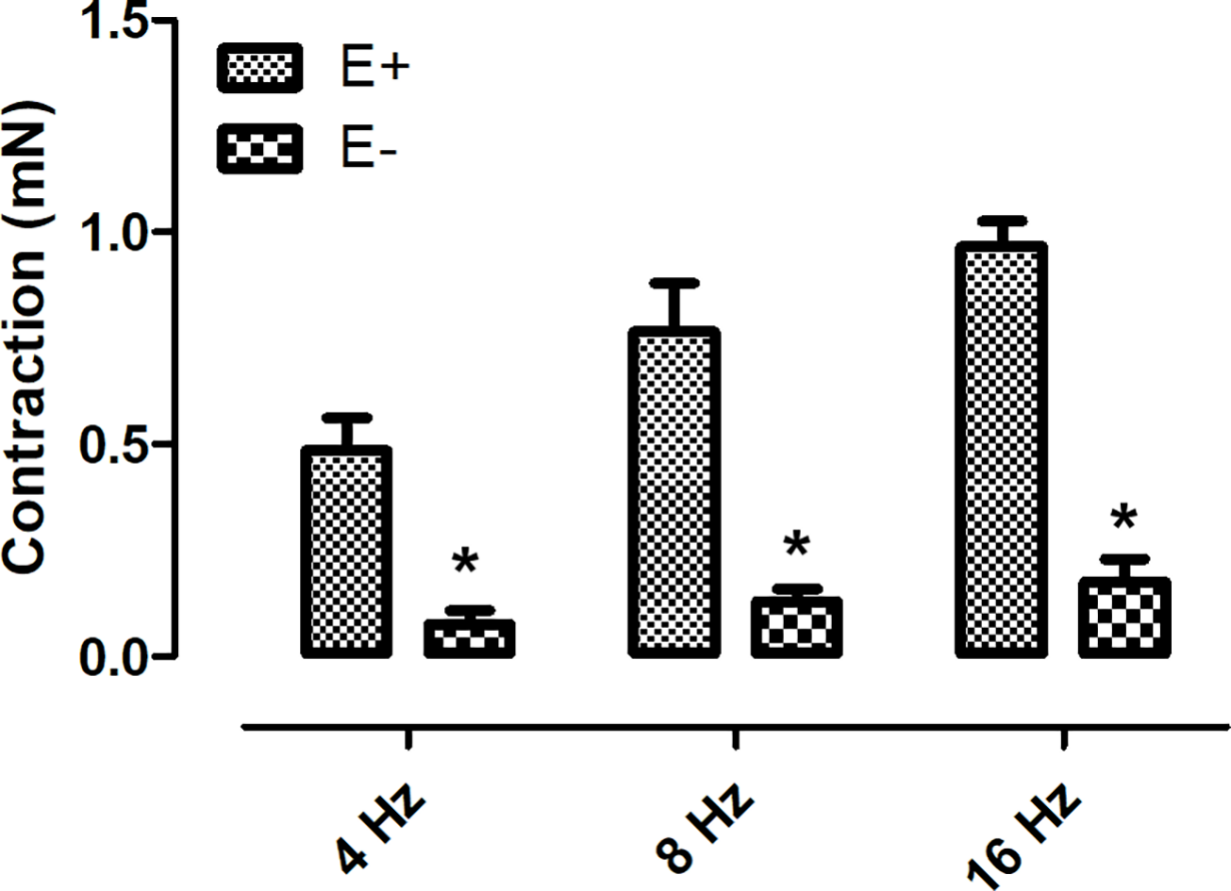
Endothelium removal reduced the EFS-induced contraction in *Panterophis guttatus* aortic rings. Data are expressed as mean ± S.E.M. *P<0.05 Vs control (unpaired Student’s t test).

**Fig 6:**
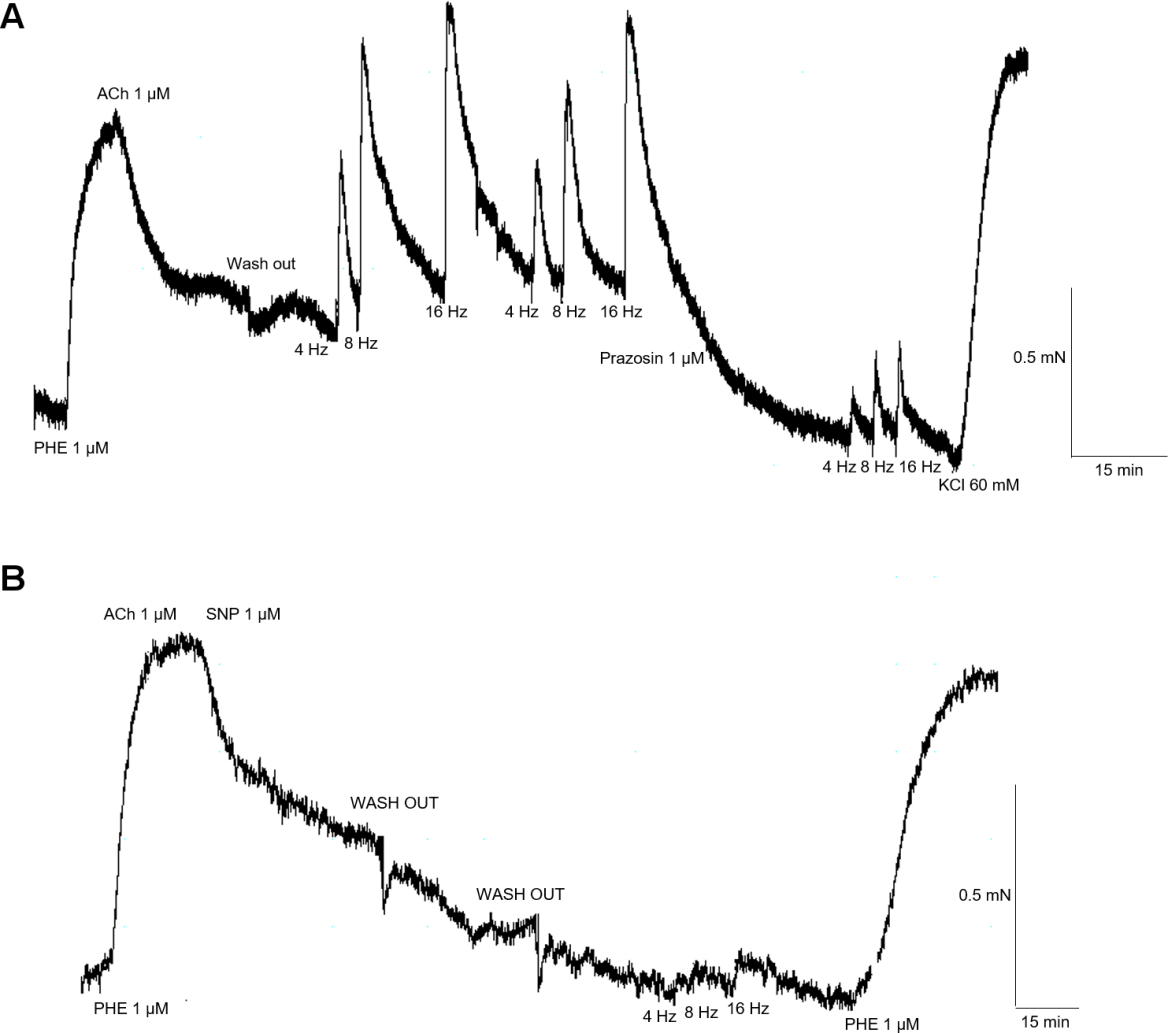
**Representative illustration of EFS-induced contractions of *Pantherophis guttatus* aortic rings with endothelium preserved (A) and denuded (B)**.

The removal of endothelium almost abolished the EFS-induced contractions in *Pantherophis guttatus* aortic rings (Fig 5 and Fig 6) (p<0.05; unpaired t-test) (n = 3, for each group).

## Discussion

These results confirm previous observations that EFS-induced contractions of corpora cavernosa in the snake *Crotalus durissus terrificus* are catecholamine dependent and insensitive to tetrodotoxin treatment [2]. Tetrodotoxin-insensitive channels were previously described in skeletal muscles of the garter snake [10,11] but not in *Crotalus durissus* skeletal muscle [1]. Since the release of catecholamine following EFS-induced contractions in the corpus cavernosum was insensitive to sodium removal and the enzymes responsible for catecholamine synthesis identified in the endothelial cells, we have proposed that the endothelium rather than adrenergic terminals would be likely source for the catecholamines [2].

As observed in the *Crotalus durissus* aortae [7], the aortic endothelium of *Pantherophis guttatus* releases nitric oxide (NO), since the NO synthesis inhibitor blocked ACh-induced relaxations. Surprisingly, the removal of the endothelium almost abolished EFS-induced contractions. In classical experiments with mammalian vascular tissues, endothelium removal is generally followed by potentiation of the vasoconstrictor effect [12].

This raises an interesting possibility of the endothelium regulation of vascular tone by release of endothelium-produced catecholamines. Although in most higher species experimental evidence supports the coexistence of catecholamines and adrenergic innervation, in lower species such as protozoa and invertebrates, the presence of catecholamine has been established although there is little, if any data, supporting the concomitant presence of adrenergic nerves [13]. Noradrenaline and related compounds were identified in bananas [14] and in other common fruits and vegetables [15], although their role in plant physiology is not very clear. Catecholamines and L-isoproterenol substantially promote flowering in the duckweek *Lemna paucicostata*, and this effect is partially blocked by the non-selective β-adrenoceptor antagonist propranolol [16], indicating a possible functional adrenergic β-receptor in plants. Thus, it is possible that control of vascular tone by the endothelium may have preceded autonomic control. This concept for vasodilatation is widely accepted, since one of the main physiological mechanism of NO release is shear stress [17–19], but not for adrenergic control.

It is unlikely that this phenomenon is restricted to snake circulation. Tyrosine hydroxylase is present in bovine and mice endothelial cells [20]. Endothelial cells isolated from the porcine pulmonary trunk were shown in culture to synthesize and release dopamine [21]. Messenger DNA coding for tyrosine hydroxylase, aromatic L-aminoacid decaborxylase and dopamine β-hydroxylase were detected by RT-PCR in culture endothelial cells from rat mesenteric artery [21]. Although the possible physiological relevance of these findings is considered unclear at the moment, one explanation is that the importance of the sympathetic control of the microcirculation has been greatly overestimated.

In healthy humans, the sympathetic nervous system is thought to contribute importantly to basal vascular tone as assessed by pharmacological α-adrenoceptor blockade [22–24]. In spinal cord-injured individuals, the supraspinal sympathetic control of leg vascular tone is lost. Surprisingly, leg vascular resistance is increased in patients with spinal cord injury [25,26] and administration of the alpha-blocker phentolamine in these patients reduced the leg vascular resistance [27], indicating a likely non-adrenergic nervous source for the catecholamines. Actually, morphological evidence of sympathetic nerve terminals in the microcirculation is very difficult to find. However, the true role of endothelium adrenergic control of the microcirculation is yet to be determined.

## Conclusion

The EFS-induced release of catecholamines from both corpus cavernosum and aorta is insensitive to TTX. The removal of endothelium abolishes EFS-induced contraction of aorta, indicating the endothelium as the source of catecholamine release.
